# CMV and EBV Co-Infection in HIV-Infected Children: Infection Rates and Analysis of Differential Expression of Cytokines in HIV Mono- and HIV–CMV–EBV Co-Infected Groups

**DOI:** 10.3390/v14081823

**Published:** 2022-08-19

**Authors:** Fizza Nazim, Hammad Afzal Kayani, Apsara Ali Nathwani, Fatima Mir, Syed Hani Abidi

**Affiliations:** 1 Department of Biological and Biomedical Sciences, Aga Khan University, Karachi 74800, Pakistan; 2 Department of Biosciences, Shaheed Zulfikar Ali Bhutto Institute of Science and Technology, Karachi 75600, Pakistan; 3 Department of Pediatrics and Child Health, Aga Khan University, Karachi 74800, Pakistan; 4 Department of Biomedical Sciences, School of Medicine, Nazarbayev University, Nur-Sultan 010000, Kazakhstan

**Keywords:** HIV mono-infection, HIV/CMV/EBV co-infection, TGF-β1, IFN-γ, cytokines, mRNA expression

## Abstract

(1) Background: CMV and EBV co-infections can affect the HIV disease progression by modulating the immune system. The disease dynamics can differ in HIV-positive adults and children. In Pakistan, HIV is rapidly expanding, especially in children; however, the prevalence of CMV and EBV co-infection and the effect on immune modulation in HIV-positive children are not known. This study aimed to bridge this gap by estimating the rate of active CMV and EBV co-infection in HIV-positive children, followed by the analysis of differential expression of cytokines in HIV mono- and HIV/CMV/EBV co-infected children. (2) Methods: DNA samples from 319 HIV-positive children, previously recruited as part of a study to investigate the HIV outbreak in Larkana, Pakistan, in 2019, were screened for CMV and EBV through qPCR. Subsequently, differences in HIV viral loads and CD4 counts were analyzed between the HIV mono- and HIV/CMV/EBV co-infected groups. The RNA samples were used to determine the differential expression of both pro- and anti-inflammatory cytokines in the mono- and co-infected groups using RT-qPCR, while unpaired T-test and Pearson correlation test were applied to, respectively, analyze the differential cytokine expression and correlation between cytokine in the two groups. (3) Results: Of 319 samples, the rate of active EBV and CMV co-infection in HIV-positive children was observed in 79.9% and 38.9%, respectively. A significant difference was observed in HIV viral load between HIV mono- and co-infected groups. IFN-γ expression was found to be lower in the HIV mono-infected group, while higher in all other three co-infected groups. Meanwhile, mRNA expression of TGF-β1 was found to be lower in HIV mono- and HIV–CMV–EBV co-infected groups, while higher in HIV–CMV and HIV–EBV co-infected groups. IFN-γ and IL-2 exhibited a significant positive correlation in all except HIV–CMV co-infected group. (4) Conclusions: The study suggests that the presence of EBV/CMV co-infection can affect the HIV viral loads and expression of certain cytokines (IFN-γ and TGF-β1), which may affect the HIV disease dynamics in infected children.

## 1. Introduction

Since the advent of antiretroviral therapy (ART), the span and quality of life of HIV-infected patients has significantly improved. However, HIV-related morbidity and mortality are still high in certain countries, especially in the low- and middle-income countries (LMIC), primarily because of low ART coverage, poor adherence to ART, immune dysfunction, inflammation, and chronic co-infections, such as Epstein–Barr virus (EBV), cytomegalovirus (CMV) infections, etc. [1,2].

The global prevalence of EBV and CMV ranges from 60 to 100%, varying largely between developed and developing countries [3,4,5,6,7]. Immunocompromised, HIV-positive individuals are especially susceptible to infection or activation of these viruses [8,9,10]. Co-infection frequency of CMV and EBV with HIV is high and there is convincing evidence reflecting a significant role of these viruses in HIV disease progression [11]. For example, one study reported that, in HIV-infected individuals with CMV seropositive status, the risk of progression to AIDS was about two times more rapidly than in CMV-seronegative HIV-infected individuals [12]. Similarly, EBV infection has also been identified as a factor associated with morbidities in HIV-infected patients [13]. In HIV-positive patients, factors such as immunological dysfunction, persistent immune activation, and T-cell receptor (TCR) repertoire loss are significantly associated with reactivation of EBV and the development of EBV-associated pathologies, such as B-cell lymphoma [14]. 

Cytokines, both anti- and proinflammatory, are the key modulators of HIV disease progression [15]. Cytokines known to induce the spread of HIV include TNF-α, TNF-β, IL-1, and IL-6, which promotes viral replication in T cells [16,17,18]. The specific mechanism(s) by which cytokines affect HIV disease progression in the case of CMV/EBV co-infection remains poorly understood. It has previously been documented that secretion of T-helper type 1 (Th 1) cytokines, such as interleukin (IL-2) and interferon-gamma (IFN-γ), is reduced during HIV-1 infection, although production of T helper 2 (Th 2) cytokines, such as IL-4, IL-10, IL-1β, IL-6, tumor necrosis factor (TNF-α), and TGF-β1, is elevated [19,20]. A previous study examining CMV and HIV co-infection found higher IL-1 and -8 levels in co-infected individuals, as compared to only HIV-infected individuals [21]. However, not much is known about the differential expression of these key cytokines in HIV–CMV and/or HIV–EBV co-infections, especially in children. 

In Pakistan, HIV exists as a concentrated epidemic in several key population groups, such as people who inject drugs (PWID), men who have sex with men (MSM), etc. [22,23,24]. Unfortunately, very little is known about the HIV epidemic in the pediatric population, and, before the April 2019 outbreak and over 13 years, only 1041 children were registered for HIV treatment [25]. The 2019 HIV outbreak in Larkana exposed a large number of HIV-positive children who acquired HIV through contaminated needles [25,26,27,28,29,30]. However, nothing is known about viral (EBV/CMV) co-infections and associated immunological changes among these children. The aim of this study was, therefore, to investigate the rate of active EBV and CMV infection in the retrospectively collected HIV samples from the 2019 Larkana outbreak, followed by a comparative assessment of the expression of eight key cytokines in HIV mono- and CMV/EBV co-infected groups.

## 2. Materials and Methods

### 2.1. Study Design and Samples 

This was a retrospective cross-sectional study, conducted on a total of 319 samples previously collected from HIV-positive children from Larkana, Pakistan, between April and July 2019 as part of the 2019 HIV outbreak investigation [25,26,27,29]. The study was conducted after obtaining written informed assent from participants and informed consent from the parents/guardians. This study was approved by the Aga Khan University Ethical Review Committee (ERC# 2021-6809-20076 and 2019-1536-4200). The data relating to HIV viral loads and CD4 counts (performed at the time of sample collection in 2019) were obtained from patients’ medical records. At the time of sample collection, most of the participants (84.4%) were receiving ART, for a median of 41 days, with the treatment regimen comprising nevirapine, lamivudine, and zidovudine, while 15.6% were ART naïve [29]. Prior to sample collection, the HIV status of the participants was unknown. 

### 2.2. Nucleic Acid (DNA and RNA) Extraction and cDNA Synthesis

DNA/RNA were previously extracted from PBMCs using Qiagen’s QIAamp DNA blood mini kit and TRIzol reagent (Gibco, Invitrogen Corporation, Waltham, MA, USA), respectively, as per the manufacturer’s instructions [24]. The DNA and RNA samples were stored at −20 °C and −80 °C, respectively, until further processing. Approximately 500 ng of RNA was reverse transcribed by using OneScript^®^ Plus cDNA Synthesis Kit, ABM, Canada (Cat#G236), as per the manufacturer’s instructions. 

### 2.3. Quantitative PCR for Detection of CMV and EBV

The DNA samples were used to detect CMV and EBV using a q-PCR strategy. For the detection of CMV and EBV, 10 μL reaction mixture was prepared using the following recipe: 1 μL of DNA, 0.3 μL (0.3 μM) of each primer (forward and reverse), 5 μL of BlasTaq™ 2X PCR master mix (ABM, Canada, cat# G891), and Nuclease Free Water to make up the volume. The sequence of forward and reverse primers for EBV were: 5′-GCTTAGCCAGTAACCCAGCACT-3′ and 5′-TGCTTAGAAGGTTGTTGGCATG-3′, respectively, while the sequence of forward and reverse primers for CMV were: 5′-GCGCGTACCGTTGAAAGAAAAGCATAA-3′ and 5′-TGGGCACTCGGGTCTTCATCTCTTTAC-3′, respectively. CFX96™ Real-Time PCR System (BIO-RAD, USA) was used to perform the qPCR reaction, using the following thermal-cycling protocol: 10 min at 95 °C, 40 cycles of 15 s at 95 °C, and 30 s at 58 °C. Melt curve (55–95 °C) analysis was performed at the end of 40 cycles to confirm the specificity of the PCR products. All reactions were run in triplicate. β-actin was used as a housekeeping gene. Additionally, non-template control (NTC) was included on each plate for each primer’s control. The B95.8 (extracted DNA) and known CMV-positive DNA sample served as a positive control for EBV and CMV, respectively. 

### 2.4. Quantitative PCR for Assessment of Cytokine Expression in HIV Mono- and CMV/EBV Co-Infected Samples

Based on EBV and CMV screening, the samples were categorized into four groups: (a) HIV+ (mono-infected), (b) HIV+/CMV+, (c) HIV+/EBV+, and (d) HIV+/CMV+/EBV+ (co-infected). For cytokine analysis, all samples from HIV+/CMV+ and HIV+/CMV+/EBV+ groups were tested, while 50/58 HIV+ and 50/255 HIV+/EBV+ samples were tested based on 18–80% incidence (derived from case incidence in this study), 80% power, and 95% confidence interval, which represents the true population characteristics [31]. 

For cytokine analysis, 10 μL sample reaction mix was prepared using the following recipe: 5 μL of BlasTaq™ 2X PCR Master Mix (cat# G891, ABM, Canada), 1 μL of primer mix (10 μM forward and reverse primers; Table 1), 1 μL cDNA, and 2 μL of nuclease-free H_2_O. The qPCR reaction was performed using the following thermal-cycling protocol: 3 min at 95 °C, 40 cycles of 15 s at 95 °C, and 58 °C for 30 s. Melt curve (55–95 °C) analysis was performed at the end of 40 cycles to confirm the specificity of the PCR products. All reactions were run in duplicate. β-actin was used as a housekeeping gene to normalize the expression of cytokines. Additionally, non-template/non-primer control (NTC/NPC) was included on each plate as a negative control for each primer. This strategy has been optimized previously in our laboratory [32,33,34]. The cytokine expression was determined using the ΔCt method [35,36].

### 2.5. Statistical Analysis

An unpaired T-test was applied to measure the significant difference in the mean HIV viral load, CD4+ cell count, and cytokine gene expression between each of the four groups. Similarly, Pearson correlation was employed to analyze the correlation between cytokine expressions in each group. SPSS version 20 was used for all statistical analyses, where a *p* < 0.05 was considered to be significant. 

## 3. Results

### 3.1. Study Subjects and EBV and CMV Status

Out of the 319 subjects, 18% (n = 58) were found to be HIV mono-infected, while 79.9% (n = 255), 38.9% (n = 124), and, out of these, 18.5% (n = 59) were found co-infected with EBV, CMV, and both CMV and EBV, respectively. 

The highest mean HIV viral load was observed in the HIV–EBV co-infected group, while the lowest mean HIV viral (341 copies/mL) load was observed in CMV co-infected patients (Table 2). The CD4 count in all four groups ranged from 1094 to 1144 cells/ mm^3^ (Table 2). The statistical analysis showed no significant difference in CD4 count in all four groups.

### 3.2. Differential Expression of Cytokines in HIV Mono- and Co-Infected Groups

**Figure 1:**
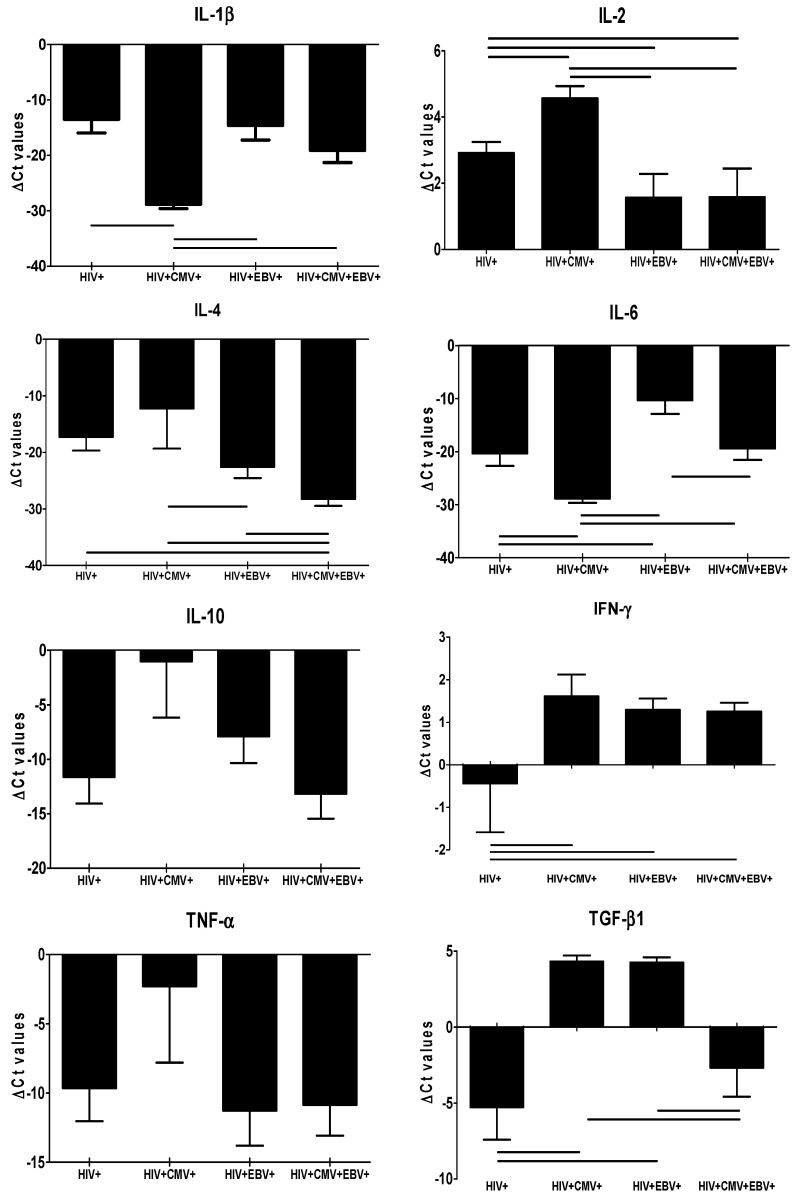
Mean ΔCt values of different cytokines in all four groups. The ΔCt values for different pro- and anti-inflammatory cytokines in HIV mono-infected and HIV–CMV, HIV–EBV, and HIV–CMV–EBV co-infected groups are shown. Solid lines above bars indicate a statistically significant difference (*p* < 0.05).

The overall analysis showed decreased mRNA expression of IL−1β, −4, −6, −10, and TNF−α, while there was increased IL−2 expression in all four groups (Figure 1 and Supplementary File). Analysis of differential cytokine mRNA expression showed IFN−γ to be significantly decreased in the HIV mono-infected group (−0.82 ± 8.12), while it was increased in all other three co-infected groups (HIV+/CMV+ = 1.61 ± 1.25; HIV+/EBV+ = 1.30 ± 1.85; and HIV+/CMV+/EBV+ = 1.25 ± 1.58; Figure 1). Similarly, the expression of TGF-β1 was found to be significantly decreased in HIV mono-infected (−5.65 ± 14.92) and HIV–CMV–EBV co-infected (−2.68 ± 14.59) groups, while it was increased in HIV–CMV (4.32 ± 0.98) and HIV–EBV (4.25 ± 2.37) co-infected groups.

**Figure 2:**
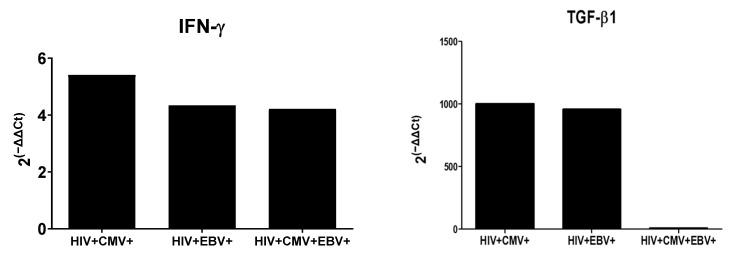
Fold change 2^(^^−^^ΔΔCt)^ analysis of IFN-γ and TGF-β1 in EBV/CMV co-infected groups as compared to HIV mono-infected group.

The 2^(^^−^^ΔΔCt)^ analysis showed the expression of IFN-γ to be ~5-, ~4-, and ~4-fold higher in HIV/CMV, HIV/EBV, and HIV/CMV/EBV co-infected groups, respectively, as compared to mono-infected groups. Similarly, the TGF-β1 expression was found to be ~1000-, ~956-, and ~8-fold higher in HIV/CMV, HIV/EBV, and HIV/CMV/EBV co-infected groups, respectively, as compared to mono-infected groups (Figure 2).

### 3.3. Correlation between Differentially Expressed Cytokines in HIV Mono- and Co-Infected Groups

Since IFN-γ and TGF- β1 were found to be differentially expressed in mono- and co-infected groups, in the next step, we determined the correlation between the gene expression of IFN-γ and TGF- β1 and other cytokines in all four groups independently in order to identify the influence (positive or negative) of one cytokine to another [37,38]. In the HIV mono-infected group, a significant positive correlation was observed between IFN-γ and IL-2 (r = 0.37, *p* = 0.008), and IFN-γ and IL-10 (r = 0.30, *p* = 0.033). Similarly, expression of TGF-β1 and IL-4 (r = 0.63, *p* = 0.00), TGF-β1 and IL-10 (r = 0.54, *p* = 0.00), and TGF-β1 and TNF-α (r = 0.48, *p* = 0.00) were also positively correlated (Table 3). 

In the HIV+/EBV+ group, a significant positive correlation was observed between IFN-γ and IL-1β (r = 0.49, *p* = 0.00), IFN-γ and IL-2 (r = 0.68, *p* = 0.00), IFN-γ and IL-10 (r = 0.49, *p* = 0.00), and IFN-γ and TNF-α (r = 0.535, *p* = 0.00). Similarly, TGF-β1 and IFN-γ (r = 0.32, *p* = 0.023), and TGF-β1 and IL-2 (r = 0.37, *p* = 0.008) were also positively correlated (Table 3). 

In the HIV/CMV/EBV triple co-infected group, IFN-γ and IL-2 (r = 0.501, *p* = 0.00), IFN-γ and IL-4 (r = 0.45, *p* = 0.00), TGF-β1 and IFN-γ (r = 0.501, *p* = 0.00), TGF-β and IL-1β (r = 0.37, *p* = 0.003), TGF-β and IL-4 (r = 0.38, *p* = 0.002), TGF-β and IL-6 (r = 0.40, *p* = 0.002), and TGF-β and IL-10 (r = 0.37, *p* = 0.003) were positively correlated (Table 3). No significant positive or negative correlation was observed in the HIV/CMV group. 

## 4. Discussion

In this study, we investigated the rate of active EBV and CMV infection in the samples collected from HIV-positive children during the 2019 Larkana outbreak. Subsequently, we performed a comparative assessment of cytokine expression in HIV mono-infected and HIV/CMV/EBV co-infected samples. 

The majority (80%) of the HIV-positive children were found to be co-infected with EBV, while ~40% with CMV, and, out of these, 18.5% with both EBV and CMV. Variable prevalence of CMV in HIV-positive patients has been reported from different parts of the world, for example, 32.4% (adults; age: 19.5–41.5 years) in India [39], 94% (both children and adults; age: 3–58 years) in Iran [40], 12.1% (infants) in Nigeria [41], 79% (age: 6-week-old infants) in Zimbabwe [42], and 10.3% (neonates) in France [43]; however, contrary to reported prevalence worldwide, we observed a CMV infection rate of about 40% in HIV-positive children from our cohort. Conversely, a high rate (80%) of EBV co-infection was observed in our cohort, which matches the rates reported in North India (62%) [44] and the Netherlands (64%) [45], while it is higher than the rates reported in Kenya (38.6%) [46]. Previous studies from the US and Zimbabwe have reported co-infection of CMV and EBV with HIV separately [47,48]. A research study conducted on Kenyan infants (HIV-infected) observed 93.9% of the infants to be simultaneously co-infected with CMV and EBV, pointing to common transmission risk factors [42]. In Pakistan, few studies have reported HIV prevalence in children [23,25]; however, to the best of our knowledge, no study has reported the rate of active CMV and EBV infection in HIV-positive children. Our study, therefore, is the first report to describe high EBV and CMV infection rates in HIV-infected Pakistani children. 

In the next step, we analyzed the differences in HIV viral load and CD4 count between HIV mono- and co-infected groups. The CD4 counts were comparable between the mono- and co-infected groups; however, the HIV viral load was found to be significantly lower (*p*-value < 0.0001) among HIV–CMV and HIV–CMV–EBV as compared to the HIV mono-infected group. Santos et al. reported a high prevalence of EBV in HIV-seropositive individuals and showed that HIV viral load was a key factor for EBV (type 1 and 2) co-infection [49]. Similarly, another study found HIV viral load to be the risk factor for CMV co-infection [50]. It is speculated that, during herpesvirus/HIV co-infection, CD4 T cell proliferation increases, thereby expanding the target cell type susceptibility to HIV infection, resulting in a high HIV viral load [51,52,53,54]. 

To date, limited studies have analyzed the differential expression of cytokines in HIV-infected children co-infected with CMV and EBV. Therefore, in this study, we investigated the differential expression of cytokine transcripts (both pro- and anti-inflammatory) in HIV mono-infected and co-infected with CMV and EBV children. We found the expression level of IFN-γ to be significantly decreased in HIV mono-infection groups, while it was increased in HIV co-infected children. It is hypothesized that infants/neonates have reduced IFN-γ-producing cells and IFN-γ levels [55], which, however, increases with age in HIV-infected patients [56]. IFN-γ is essential for the regulation of chronic and latent infection of herpes virus (alpha, beta, and gamma) [57,58,59]. Infection with CMV, in particular, has been shown to induce a significant expression of IFN-γ and other Th1 cytokines by effector CMV-specific effector T cells [60]. Similarly, studies have also shown a correlation between increased IFN-γ levels and EBV reactivation [61,62]. Limited studies have reported the expression of IFN-γ in HIV and CMV/EBV co-infection. The decreased IFN-γ observed in the mono-infected group may be attributed to the acute or early chronic phase of HIV infection [63], while the presence of herpes virus co-infection may lead to increased IFN-γ and Th-1 response, which is also supported by correlation analysis, where IFN-γ expression correlated with IL-2 and TGF- β1. 

Interestingly, in the HIV mono- and HIV–CMV–EBV triple co-infection group, TGF-β1 expression levels were significantly lower, but they were higher in HIV–CMV and HIV–EBV co-infection groups. TGF-β1 production in HIV infection has varied kinetics depending on the cell type, which raises the possibility that TGF-β1 might play both positive and deleterious functions during infection. TGF-β1 has shown to be rapidly and systemically generated after acute HIV-1 infection and is maintained at an elevated level [64]. The concentration of TGF-β1 is linked with HIV disease progression; as the disease progresses, the TGF-β1 levels also increase [65,66]. CMV infection increases immunological tolerance in an immunocompromised environment by boosting TGF-1 transcription and release and suppressing cytotoxic Th1 cells [67,68,69]. Similarly, EBV cell lines generate TGF-1 and are resistant to TGF-1-mediated apoptosis and growth inhibition, which aids in the proliferation of EBV-infected cells [70]. It has been reported that other co-infection with HIV causes a significant increase in TGF-β1 levels [71,72]. Our study also showed an increase in TGF-β1 levels in the HIV/CMV/EBV co-infected group, which may enhance the CMV/EBV infection [73]. 

We identify certain limitations of this study. Firstly, the sample size of HIV/CMV co-infected patients was very low in this cohort. A comparison with a higher number of HIV/CMV samples might affect the outcomes related to the markers of HIV disease progression (CD4 count and viral loads) and/or cytokine expression. Secondly, due to the availability of only DNA and RNA samples (and the absence of serum/plasma samples), serological detection of CMV and EBV could not be performed. Due to the same reason, we could only analyze the mRNA expression of cytokines and not the protein expression. However, it is important to note that numerous studies have investigated changes in cytokine expression at the mRNA levels only and have reported differential cytokine gene expression [74,75]. 

## 5. Conclusions

In conclusion, the high co-infection with CMV and EBV in HIV-positive children may affect the HIV viral loads and expression of certain cytokines (IFN-γ and TGF-β1), which may affect the HIV disease dynamics. Further mechanistic understanding of the involvement of herpes viruses in HIV-positive children may provide insights into disease pathogenesis. 

## Figures and Tables

**Table 1:** Name of target genes and respective primer sets used to quantify mRNA levels in qPCR.

Gene	Forward Primer (5′ to 3′)	Reverse Primer (5′ to 3′)
Β-actin	GCGCGGCTACAGCTTCA	CTCCTTAATGTCACGCACGAT
IL-1β	ATGATGGCTTATTACAGTGGCAA	GTCGGAGATTCGTAGCTGGA
IL-2	GAAGATCGTCATGGGAAGAAGC	CGGGTATTTATAGTGGCATGGG
IL-4	CCAACTGCTTCCCCCTCTG	TCTGTTACGGTCAACTCGGTG
IL-6	ACTCACCTCTTCAGAACGAATTG	CCATCTTTGGAAGGTTCAGGTTG
IL-10	GACTTTAAGGGTTACCTGGGTTG	TCACATGCGCCTTGATGTCTG
IFN-γ	TCGGTAACTGACTTGAATGTCCA	TCGCTTCCCTGTTTTAGCTGC
TNF-α	GAGGCCAAGCCCTGGTATG	CGGGCCGATTGATCTCAGC
TGF-β1	CAATTCCTGGCGATACCTCAG	GCACAACTCCGGTGACATCAA

**Table 2:** Virological and clinical features of HIV mono- and HIV/CMV/EBV co-infected groups. The table shows the average Ct values for EBV and CMV in co-infected groups, as well as CD4 count and HIV viral loads in mono- and co-infected groups The *p*-values are given in the last column, where a significant *p*-value (*p* < 0.05) is indicated by *.

Variables	HIV+	HIV/CMV/EBV+	HIV/CMV+	HIV/EBV+	*p*-Value
CMV q-PCR (average Ct)	-	34.94	33.71	-	-
EBV q-PCR (average Ct)	-	33.37	-	34.52	-
Mean HIV viral load	42,396.2	24,154.30	341.25	147,030.21	0.001–0.002 *
Mean CD4 count	1144	1133	1105.5	1093.92	0.6–0.9

**Table 3:** Correlation of eight cytokines in HIV mono- and co-infected groups. Each column shows the R-value (the coefficient of correlation). Correlations with *p* < 0.05 are indicated with * and *p* < 0.01 are indicated with **.

**HIV+ Mono-Infected Group**								
**Cytokines**	**IL-1β**	**IL-2**	**IL-4**	**IL-6**	**IL-10**	**IFN-γ**	**TNF-α**	**TGF-β1**
IFN-γ	0.20	0.37 **	−0.02	0.14	0.30 *	-	0.17	0.14
TGF-β	0.06	0.11	0.63 **	0.20	0.54 **	0.14	0.48 **	-
**HIV+/CMV+ co-infected group**								
**Cytokines**	**IL-1β**	**IL-2**	**IL-4**	**IL-6**	**IL-10**	**IFN-γ**	**TNF-α**	**TGF-β1**
IFN-γ	0.43	0.71	−0.77	0.43	0.03	-	−0.09	0.43
TGF-β	−0.31	0.14	−0.37	−0.31	0.26	0.43	−0.20	-
**HIV+/EBV+ co-infected group**								
**Cytokines**	**IL-1β**	**IL-2**	**IL-4**	**IL-6**	**IL-10**	**IFN-γ**	**TNF-α**	**TGF-β1**
IFN-γ	0.49 **	0.68 **	0.22	0.24	0.49 **	-	0.53 **	0.32 *
TGF-β1	0.22	0.37 **	0.03	0.26	0.10	0.32 *	0.01	-
**HIV+/CMV+/EBV+ co-infected group**								
**Cytokines**	**IL-1β**	**IL-2**	**IL-4**	**IL-6**	**IL-10**	**IFN-γ**	**TNF-α**	**TGF-β1**
IFN-γ	0.14	0.50 **	0.45 **	0.28 *	0.23	-	−0.20	0.50 **
TGF-β	0.37 **	0.18	0.38 **	0.40 **	0.37 **	0.50 **	0.15	-

## Data Availability

The data are available within the manuscript or its Supplementary Files.

## Supplementary Materials

The following supporting information can be downloaded at: https://www.mdpi.com/article/10.3390/v14081823/s1, Supplementary File.
